# Supplementary material for: Freshwater hydrozoan blooms alter activity and behaviour of territorial cichlids in Lake Tanganyika
Source: R Soc Open Sci. 2019 Nov 6;6(11):191053. doi: 10.1098/rsos.191053 (PMC6894582; doi:10.1098/rsos.191053)
Supplement: Tukey pairwise contrasts for V. moorii behaviours in presence/absence of jellyfish and offspring [file rsos191053supp1.docx]

SUPPLEMENTARY MATERIALS

**Supplementary Materials Table 1:** Results of Tukey pairwise contrasts for linear and generalized linear mixed effects models described in methods section. Results of analysis for (A) average movement activity (in body lengths travelled per second), (B) defence behaviours, (C) social affiliation behaviours, (D) feeding behaviours, and (E) body scrapes observed in each 15-minute field video. 1s and 0s in parentheses respectively indicate the presence or absence of high *Limnocnida tanganjicae* jellyfish densities or offspring at the *Variabilichromis moorii* territories. Significant *P*-values (< 0.05) are in bold.

|  | | | Est. ± SE | z-value | *P* |
| --- | --- | --- | --- | --- | --- |
| **(A) Average movement activity** | | |  |  |  |
| Jellyfish (1)  Offspring (0) | vs. | Jellyfish (1)  Offspring (1) | 0.10 ± 0.05 | 1.90 | 0.23 |
| Jellyfish (0)  Offspring (1) | vs. | Jellyfish (1)  Offspring (1) | 0.32 ± 0.05 | 6.00 | **<0.001** |
| Jellyfish (0)  Offspring (0) | vs. | Jellyfish (1)  Offspring (1) | 0.24 ± 0.05 | 4.46 | **<0.001** |
| Jellyfish (0)  Offspring (1) | vs. | Jellyfish (1)  Offspring (0) | 0.22 ± 0.05 | 4.25 | **<0.001** |
| Jellyfish (0)  Offspring (0) | vs. | Jellyfish (1)  Offspring (0) | 0.14 ± 0.05 | 2.67 | **0.04** |
| Jellyfish (0)  Offspring (0) | vs. | Jellyfish (0)  Offspring (1) | -0.08 ± 0.05 | -1.56 | 0.40 |
| **(B) Defence behaviours** | | |  |  |  |
| Jellyfish (1)  Offspring (0) | vs. | Jellyfish (1)  Offspring (1) | -0.02 ± 0.38 | -0.06 | 0.99 |
| Jellyfish (0)  Offspring (1) | vs. | Jellyfish (1)  Offspring (1) | 1.15 ± 0.37 | 3.15 | **0.009** |
| Jellyfish (0)  Offspring (0) | vs. | Jellyfish (1)  Offspring (1) | 0.42 ± 0.37 | 1.12 | 0.68 |
| Jellyfish (0)  Offspring (1) | vs. | Jellyfish (1)  Offspring (0) | 1.17 ± 0.35 | 3.32 | **0.005** |
| Jellyfish (0)  Offspring (0) | vs. | Jellyfish (1)  Offspring (0) | 0.44 ± 0.36 | 1.21 | 0.62 |
| Jellyfish (0)  Offspring (0) | vs. | Jellyfish (0)  Offspring (1) | -0.73 ± 0.34 | -2.17 | 0.13 |

**Supplementary Materials Table 1:** Continued.

|  | | | Est. ± SE | z-value | *P* |
| --- | --- | --- | --- | --- | --- |
| **(C) Social affiliative behaviours** | | |  |  |  |
| Jellyfish (1)  Offspring (0) | vs. | Jellyfish (1)  Offspring (1) | 2.20 ± 0.87 | 2.54 | 0.051 |
| Jellyfish (0)  Offspring (1) | vs. | Jellyfish (1)  Offspring (1) | 0.90 ± 0.91 | 0.99 | 0.74 |
| Jellyfish (0)  Offspring (0) | vs. | Jellyfish (1)  Offspring (1) | 2.87 ± 0.86 | 3.34 | **0.004** |
| Jellyfish (0)  Offspring (1) | vs. | Jellyfish (1)  Offspring (0) | -1.30 ± 0.56 | -2.34 | 0.085 |
| Jellyfish (0)  Offspring (0) | vs. | Jellyfish (1)  Offspring (0) | 0.67 ± 0.48 | 1.41 | 0.48 |
| Jellyfish (0)  Offspring (0) | vs. | Jellyfish (0)  Offspring (1) | 1.97 ± 0.54 | 3.64 | **0.001** |
| **(D) Feeding behaviours** | | |  |  |  |
| Jellyfish (1)  Offspring (0) | vs. | Jellyfish (1)  Offspring (1) | 0.17 ± 0.68 | 0.25 | 1.00 |
| Jellyfish (0)  Offspring (1) | vs. | Jellyfish (1)  Offspring (1) | 0.97 ± 0.66 | 1.47 | 0.46 |
| Jellyfish (0)  Offspring (0) | vs. | Jellyfish (1)  Offspring (1) | 1.02 ± 0.66 | 1.54 | 0.41 |
| Jellyfish (0)  Offspring (1) | vs. | Jellyfish (1)  Offspring (0) | 0.79 ± 0.64 | 1.24 | 0.60 |
| Jellyfish (0)  Offspring (0) | vs. | Jellyfish (1)  Offspring (0) | 0.85 ± 0.64 | 1.32 | 0.55 |
| Jellyfish (0)  Offspring (0) | vs. | Jellyfish (0)  Offspring (1) | 0.05 ± 0.61 | 0.09 | 1.00 |
| **(E) Body scrape behaviours** | | |  |  |  |
| Jellyfish (1)  Offspring (0) | vs. | Jellyfish (1)  Offspring (1) | 0.34 ± 0.90 | 0.38 | 0.98 |
| Jellyfish (0)  Offspring (1) | vs. | Jellyfish (1)  Offspring (1) | 0.12 ± 0.90 | 0.14 | 1.00 |
| Jellyfish (0)  Offspring (0) | vs. | Jellyfish (1)  Offspring (1) | 0.07 ± 0.91 | 0.07 | 1.00 |
| Jellyfish (0)  Offspring (1) | vs. | Jellyfish (1)  Offspring (0) | -0.22 ± 0.85 | -0.26 | 0.99 |
| Jellyfish (0)  Offspring (0) | vs. | Jellyfish (1)  Offspring (0) | -0.28 ± 0.86 | -0.32 | 0.99 |
| Jellyfish (0)  Offspring (0) | vs. | Jellyfish (0)  Offspring (1) | -0.06 ± 0.85 | -0.07 | 1.00 |
